# Electrical vagus nerve stimulation is a promising approach to reducing pulmonary complications after an esophagectomy: an experimental rodent model

**DOI:** 10.1007/s12026-024-09523-3

**Published:** 2024-07-31

**Authors:** Henricus J. B. Janssen, Tessa C. M. Geraedts, Laura F. C. Fransen, Ingrid van Ark, Thea Leusink-Muis, Gert Folkerts, Johan Garssen, Jelle P. Ruurda, Grard A. P. Nieuwenhuijzen, Richard van Hillegersberg, Misha D. P. Luyer

**Affiliations:** 1 Department of Surgery, Catharina Hospital, Michelangelolaan 2, 5623 EJ Eindhoven, The Netherlands; 2 Division of Pharmacology, Department of Pharmaceutical Sciences, Faculty of Science, Utrecht University, Utrecht, The Netherlands; 3 Department of Surgery, University Medical Center Utrecht, Utrecht University, Utrecht, The Netherlands; 4 Nutricia Research, Immunology, Utrecht, The Netherlands; 5 Department of Electrical Engineering, University of Technology Eindhoven, Eindhoven, The Netherlands

**Keywords:** Esophagectomy, Pulmonary complications, Vagus nerve stimulation, Vagus nerve, Vagotomy, Inflammation

## Abstract

After esophagectomy, an imbalanced inflammatory response increases the risk of postoperative morbidity. The vagus nerve modulates local and systemic inflammatory responses, but its pulmonary branches are transected during esophagectomy as part of the oncological resection, which may account for the high incidence of postoperative (pulmonary) complications. This study investigated the effect of electrical vagus nerve stimulation (VNS) on lipopolysaccharide (LPS)-induced lung injury in rats. Rats (*n* = 60) were randomly assigned to a non-vagotomy or cervical vagotomy group, with VNS or without (NOSTIM). There were four non-vagotomy groups: NOSTIM and bilateral VNS with 100, 50, or 10 µA. The four vagotomy groups were NOSTIM and VNS with fixed amplitude (50 µA) bilaterally before (VNS-*50*-*before*) or after bilateral vagotomy (VNS-*50*-*after*), or unilaterally (left) before ipsilateral vagotomy (VNS-*50*-*unilaterally*). LPS was administered intratracheally after surgery. Pulmonary function, pro-inflammatory cytokines in serum, broncho-alveolar lavage fluid (BALF), and histopathological lung injury (LIS) were assessed 180 min post-procedure. In non-vagotomized rats, neutrophil influx in BALF following intra-tracheal LPS (mean 30 [± 23]; *P* = 0.075) and LIS (mean 0.342 [± 0.067]; *P* = 0.142) were similar after VNS-*100*, compared with NOSTIM. VNS-*50* reduced neutrophil influx (23 [± 19]; *P* = 0.024) and LIS (0.316 [± 0.093]; *P* = 0.043). VNS-*10* reduced neutrophil influx (15 [± 6]; *P* = 0.009), while LIS (0.331 [± 0.053]; *P* = 0.088) was similar. In vagotomized rats, neutrophil influx (52 [± 37]; *P* = 0.818) and LIS (0.407 [SD ± 0.037]; *P* = 0.895) in VNS-*50*-*before* were similar compared with NOSTIM, as well as in VNS-*50*-*after* (neutrophils 30 [± 26]; *P* = 0.090 and LIS 0.344 [± 0.053]; *P* = 0.073). In contrast, VNS-*50*-*unilaterally* reduced neutrophil influx (26 [± 10]; *P* = 0.050) and LIS (0.296 [± 0.065]; *P* = 0.005). Systemic levels of cytokines TNF-α and IL-6 were undetectable in all groups. Pulmonary function was not statistically significantly affected. In conclusion, VNS limited influx of neutrophils in lungs in non-vagotomized rats and may attenuate LIS. Unilateral VNS attenuated lung injury even after ipsilateral vagotomy. This effect was absent for bilateral VNS before and after bilateral vagotomy. It is suggested that the effect of VNS is dependent on (partially) intact vagus nerves and that the level of the vagotomy during esophagectomy may influence postoperative pulmonary outcomes.

**Supplementary Information:**

The online version contains supplementary material available at 10.1007/s12026-024-09523-3.

## Introduction

An esophagectomy is the mainstay of multimodal treatment for potentially curative esophageal cancer. However, the surgical procedure is technically challenging and still associated with substantial postoperative morbidity, despite several improvements in recent years (e.g., minimally invasive techniques, enhanced recovery after surgery (ERAS) programs, and prehabilitation) [1–5]. Particularly pulmonary complications remain the leading cause of prolonged hospital and intensive care unit stay, readmissions, and even mortality [6–8].

Although several factors have been associated with postoperative (pulmonary) complications, the exact pathogenesis is not fully understood [9–11]. During esophagectomy and its concurrent lymphadenectomy, pulmonary branches of the vagus nerve are often transected even though it has been shown that the majority can be technically spared [12, 13]. It has been hypothesized that transection of these vagus nerve branches accounts for the observed high incidence of postoperative pulmonary complications [12–14].

In previous studies, it has been shown that vagus nerve stimulation (VNS) may effectively inhibit inflammatory pathways both in experimental models [15–20] and clinical pilots [21–24] by the cholinergic anti-inflammatory pathway (CAIP). The mechanism of action of the CAIP involves a complex neural circuit. Acetylcholine (ACh) and the α7 nicotinic acetylcholine receptor (α7nAChR) are the key components of the CAIP. Inflammatory stimuli signal from the afferent branches of the vagus nerve to the nucleus tractus solitarius in the brainstem. This initiates the inflammatory reflex from the dorsal motor nucleus of the vagus nerve, triggering the anti-inflammatory response via the efferent pathway. Subsequently, ACh is released at efferent endings that bind to the α7nAChR located on macrophages [25–28]. The early release of pro-inflammatory cytokines by activated macrophages has a pivotal role in triggering the inflammatory response. Electrical, pharmacological, and nutritional stimulation of the CAIP inhibited the release of pro-inflammatory mediators in several study models [15–20, 24, 29, 30]. Although the anti-inflammatory actions of vagal efferents are well established, vagal afferent fibers—which comprise almost 80% of all vagal fibers—also possess anti-inflammatory properties; however, this is not solely mediated by vagal efferent activation [28–31].

The aim of this study was to investigate whether VNS affects local inflammation in a model of LPS-induced acute lung injury.

## Methods

### Animals

Male Sprague Dawley rats, weighing 350 to 400 g, purchased from Charles River Laboratories (Erkrath, Germany), were used. The rats were housed in a specialized laboratory animal facility (e.g., temperature- and humidity-controlled room, under a standard 12/12 h light/dark cycle). Food and water were provided ad libitum. All animal experiments were conducted in compliance with the Dutch Act on Animals Experiments for scientific purposes (Wet op de Dierproeven) and the Guidelines of the Ethical Committee on the Use of Laboratory Animals of the Utrecht University. The study was approved by the Dutch Central Authority for Scientific Procedures on Animals (CCD) under project number AVD1150020198345.

### Surgical procedure

Rats were anesthetized intraperitoneally with urethane 10% (U2500, Sigma-Aldrich, Zwijndrecht, The Netherlands) 2 g/kg in total divided over 3 doses within 30 min prior to surgery. Throughout the experiment, rectal temperate was monitored and kept between 36.5 and 37.5 °C using a heating pad and blankets. Anesthetic depth was assessed every 30 min by the absence of protective eye (corneal) reflexes and withdrawal reflexes after toe pinch. Rats were ordered in groups of four, and each rat was randomly assigned to one of the interventions (i.e., surgical procedure and vagus nerve stimulation). There were two intervention groups. A non-vagotomized control group (*n* = 32) underwent a procedure that reflects preservation of the pulmonary branches of the vagus nerve, in order to investigate whether electrical vagus nerve stimulation (VNS) attenuates local inflammation in the lung. In this group, a small cervical midline incision was made, and after division of the submaxillary glands and muscles, the vagus nerve was identified bilaterally and carefully dissected. Subsequently, rats were tracheotomized and a tracheal cannula was placed and fixed with ligatures, after which the cervical midline incision was closed. The vagotomy group (*n* = 28) underwent the same procedure as described above; however, after identification of the cervical vagus nerve(s), these were transected which reflects the transection of the vagal pulmonary branches. The cervical vagotomy was performed either bilaterally or unilaterally, to investigate whether there was a difference in outcomes after VNS following a complete and selective (i.e., unilateral) vagotomy.

### Experimental design

Both in the non-vagotomized and vagotomy group, rats were administered lipopolysaccharide (LPS) (Sigma-Aldrich, Zwijndrecht, The Netherlands) intratracheally post-procedure through the previously placed tracheal cannula to induce acute lung injury. As LPS dose-dependently increases inflammation, a dose of 0.3 µg/kg was used since we hypothesized based on our previous experiments that intra-tracheal administration of excessive exogenous LPS could exaggerate the inflammatory response, thereby limiting the modulatory effect of vagus nerve stimulation and resulting in more premature deaths especially in vagotomized rats [14, 32]. A pilot study was performed in a separate non-vagotomized (*n* = 22) and vagotomy (*n* = 18) group to investigate whether LPS sufficiently increased the influx of neutrophils compared to saline (0.9% NaCl), which is shown in Supplementary Figure 1a (non-vagotomized group) and Supplementary Figure 2a (vagotomy group).

**Fig. 1:**
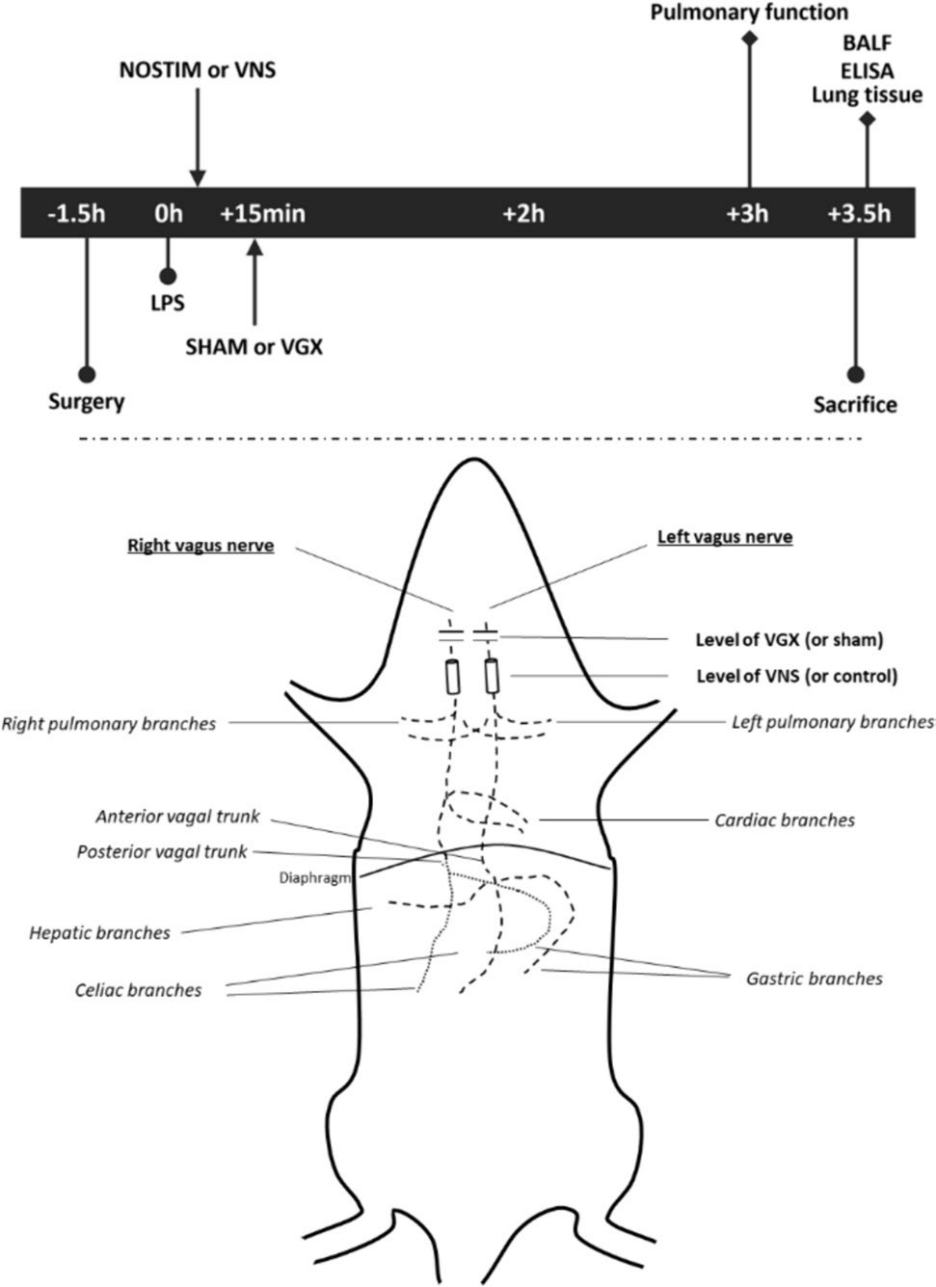
Schematic overview of the experiment. The specific surgical procedure—sham procedure versus vagotomy—and method of electrical stimulation are described in detail in the methods. Abbreviations: LPS, lipopolysaccharide; BALF, broncho-alveolar lavage fluid; SHAM, sham procedure without vagotomy (non-vagotomy); VGX, cervical vagotomy; NOSTIM, group without VNS; VNS, electrical vagus nerve stimulation

All rats underwent pulmonary function tests at 3 h and were euthanized at 3.5 h after LPS or saline by an overdose of intraperitoneally injected pentobarbital 150 mg/kg (Euthesate™, Ceva Santé Animale, Naaldwijk, The Netherlands). This timeline was shorter than our previous experiments since the pilot revealed that the combination of vagus nerve stimulation with a vagotomy was too demanding on some rats which led to premature deaths. Finally, broncho-alveolar lavage fluid (BALF), blood samples, and organ tissues were isolated and examined in accordance with the American Thoracic Society guidelines [33]. A schematic overview of the complete experiment is shown in Fig. 1.

### Electrical vagus nerve stimulation

**Fig. 2:**
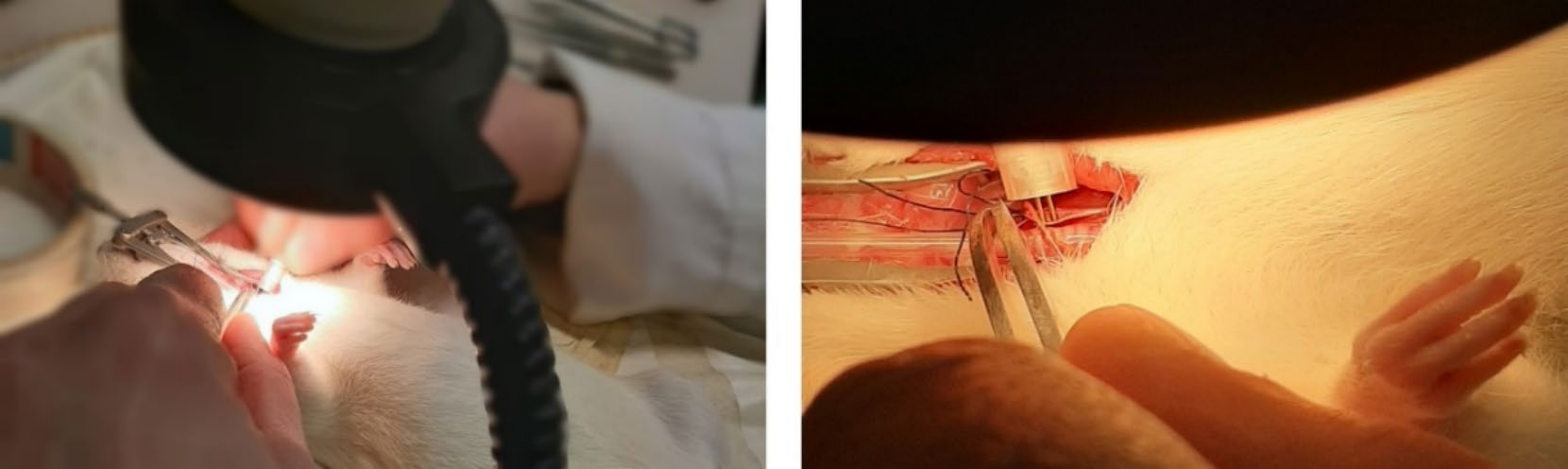
Position of rats during procedure. With the rats in the supine position, the electrode was brought gently towards the vagus nerves, after which the vagus nerves were stimulated three times in 10 min using fixed parameters: constant-current, biphasic, and charge-balanced square wave pulses (10, 50, or 100 µA/phase, 2 ms/phase at 5 Hz) for 1 min each depending on the groups as described in the methods

The non-vagotomized and vagotomy groups were also randomly assigned to either undergo VNS or be withheld stimulation (NOSTIM). Initially, after identifying the vagus nerves, a cuff—connected to an electrode—was wrapped around the vagus nerve. However, it came to our attention during the early tests that the supplied cuff malfunctioned and failed to conduct electricity. Consequently, the electrode had to be brought directly towards the cervical vagus nerves, as shown in Fig. 2. This was gently performed while avoiding excessive manipulation of the vagus nerve to prevent axonal damage. With the rat in the supine position, an electrical current was delivered to the vagus nerve(s) three times in 10 min based on fixed parameters: constant-current, biphasic, and charge-balanced square wave pulses (10, 50, or 100 µA/phase, 2 ms/phase at 5 Hz). Electric current was applied for 1 min each using the Neurolog Current Stimulus Isolator (model NL800A), Power Supply (NL900D), and Pulse Generator (NL301). These parameters were based on the experience of a research group from Amsterdam University Medical Center that provided the equipment. In the vagotomy groups, VNS was applied with a fixed amplitude (i.e., 50 µA based on early tests with the equipment). The NOSTIM groups underwent the same procedure; however, after bringing the electrode towards the vagus nerves, the system was left idle.

Altogether, the non-vagotomized (control) group comprised of four groups: a NOSTIM group and three groups with VNS (i.e., VNS-*100*, VNS-*50*, and VNS-*10)*, corresponding with the amplitudes as described above. The vagotomy group comprised of the following four groups: a NOSTIM group, a group that was bilaterally stimulated directly before bilateral vagotomy (VNS-*50*-*before*), a group that was bilaterally stimulated directly after the bilateral vagotomy (VNS-*50*-*after*), and finally a group that was unilaterally stimulated (i.e., left vagus nerve) before ipsilateral vagotomy (VNS-*50*-*unilaterally*). The latter group was designed to model the unilateral vagotomy during esophagectomy and test whether selective VNS could influence inflammation.

A pilot study was performed in a separate non-vagotomized and vagotomy group to investigate whether there could be an effect of VNS in rats that were only administered saline, as shown in Supplementary Figure 1b (non-vagotomy group) and Supplementary Figure 2b (vagotomy group).

### Pulmonary function measurement

Rats were placed in a temperature-controlled plethysmograph (body temperature was kept at 37 °C) in which they were ventilated (frequency 90 breaths/min; volume 2 mL/breath). The previously placed tracheal cannula was replaced by a small catheter and connected to a pressure transducer fixed on the plethysmograph box (EMKA Technologies, Paris, France). Transpulmonary pressure was determined by measuring pressure differences in the catheter. Airflow and tidal volume were determined using a flow transducer fixed to the body box. Increasing doses (0.37 up to 50 mg/mL, 10% puff for 10 s) of acetyl-β-methyl-choline chloride (methacholine) (Sigma-Aldrich, Zwijndrecht, The Netherlands) were administered by aerosol, generated in a nebulizer connected in-between the plethysmograph and the ventilator (EMKA Technologies, Paris, France). After the first dose of methacholine, pulmonary resistance was measured for 3 min. This procedure was repeated for all subsequent doses.

### Broncho-alveolar lavage fluid (BALF)

After the rats were sacrificed, the thoracic cavity was opened and both lungs were taken out. The right main bronchus was canulated, and the right lung was lavaged with 2 mL of pyrogen-free saline (0.9% NaCl, 37 °C) supplemented with protease inhibitor cocktail tablet (Complete Mini, Roche Diagnostics, Mannheim, Germany). The supernatant of the first mL was used for cytokine measurement. Afterwards, the right lung was lavaged two times with 2 mL saline solution (0.9% NaCl, 37 °C), and the BALF cells were centrifuged (300 g, 4 °C, 5 min). The pellets of the three lavages were pooled, and the total numbers of BALF cells were counted using a Bürker-Türk bright-line counting chamber (magnification × 100) (Karl Hecht Assistant KG, Sondheim/Rohm, Germany). For differential BALF cell counts, cytospin preparations were made and stained with Diff-Quick (Merz and Dade A.G., Düdingen, Switzerland). After coding, all cytospin preparations were evaluated by two laboratory technicians independently (I.vA and T.LM) using oil immersion microscopy (Leitz Optilux, Leica, Wetzlar, Germany). A number of macrophages, lymphocytes, and neutrophils were scored by standard morphology. At least 200 cells per cytospin preparation were counted, and the absolute number of each cell type was calculated.

### Lung histology

The left lungs were fixed with 10% formalin infusion and embedded in paraffin after fixation. Subsequently, 5-µm-thick lung sections were cut (Leica, model RM2165, Germany) and stained with hematoxylin/eosin (H&E). Photomicrographs were taken with an Eclipse E800M microscope (Nikon Instruments Inc. The Netherlands) equipped with a Nikon DXM 1200 digital Camera (Nikon Instruments Inc. the Netherlands). Sections were prepared by the pathology department of the UMC Utrecht. Histopathological lung injury was determined by two researchers (H.J. and T.G.) independently in blinded H&E sections according to the guidelines of the American Thoracic Society, in which scores range from 0 to 1, with higher scores indicating more severe lung injury [33].

### ELISA

Blood samples were obtained by cardiac puncture after rats were sacrificed. Twenty microliters of blood were used to count the total number of leukocytes, and a blood smear was made to determine the cell count of various white blood cells. The rest of the blood was centrifuged (14,000 g, room temperature, 5 min). Plasma was collected and samples were kept at − 20 °C. Subsequently, IL-6 (BMS625) and TNF-α (BMS622) were measured with a Ready-SET-Go!® ELISA kit (eBioscience, San Diego, CA, USA), and concentrations were expressed as pg/mL.

### Statistical analysis

Statistical analyses were performed using Statistical Software for the Social Sciences (SPSS) for windows version 25 (IBM Software Group). Data were tested for normal distribution using the formal Shapiro–Wilk test. Numerical data are presented as means with standard deviation (SD) or medians with interquartile range (IQR), depending on normality (Shapiro–Wilk test). One-way analysis of variance (ANOVA) with post-hoc Dunns comparison was used for statistical comparisons between baseline and other data points for multiple groups, if data were normally distributed. Similarly, a non-parametric test such as the Kruskal–Wallis with post-hoc comparison (Bonferroni) was used if data were not normally distributed. Differences between the two groups were analyzed using the *t*-test (unpaired) or Mann–Whitney test, depending on their distribution. A *P*-value < 0.05 was considered statistically significant.

## Results

### Non-vagotomy (control) group

**Fig. 3:**
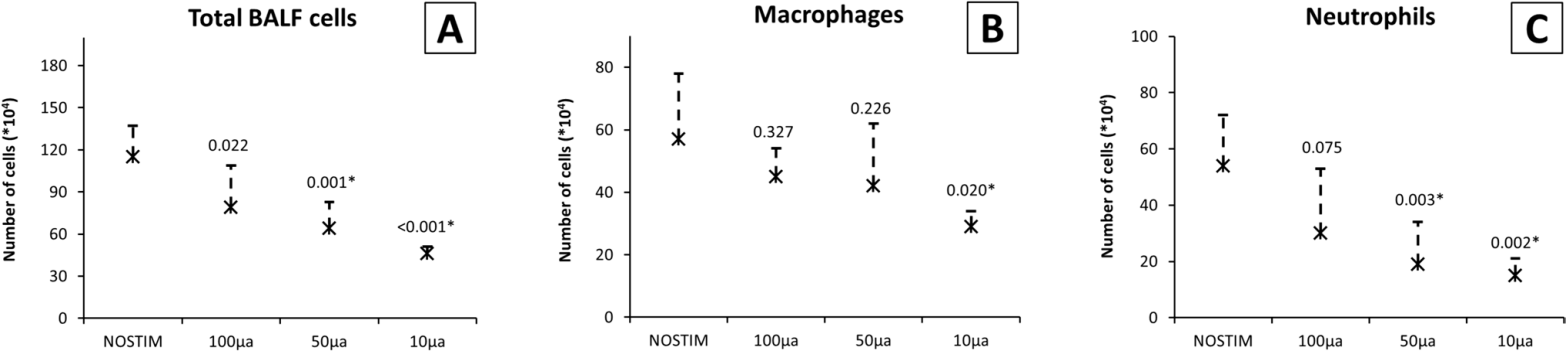
Number of inflammatory cells in BALF (× 10^4^) in non-vagotomized rats. **A** Total cell count, **B** macrophages, and **C** neutrophils. Bilateral VNS before LPS (intratracheally) significantly reduced the influx of neutrophils after 50 and 10 µA, but not 100 µA compared to NOSTIM. Cell count of macrophages was also lower after 10 µA, but not 50 or 100 µA. Values are means with standard deviation. A *P*-value of < 0.05 after post-hoc Dunn’s test was considered statistically significant. Abbreviations: BALF, broncho-alveolar lavage fluid; LPS, lipopolysaccharide; NOSTIM, group without VNS; VNS, vagus nerve stimulation

In the NOSTIM group, the total count of inflammatory cells (× 10^4^) in the BALF was mean 115 [SD ± 22], as shown in Fig. 3. Macrophage count (× 10^4^) was mean 57 [SD ± 21], and the total count of neutrophils (× 10^4^) was mean 54 [SD ± 18]. After VNS-*100*, total count of inflammatory cells was reduced (79 [± 30]; *P* = 0.022), whereas macrophage count (45 [± 9]; *P* = 0.327) and neutrophil count (30 [± 23]; *P* = 0.075) were similar compared with NOSTIM. After VNS-*50*, the total count of inflammatory cells (64 [± 19]; *P* = 0.001), as well as neutrophil count (19 [± 15]; *P* = 0.003), were reduced, while macrophage count was similar (42 [± 20]; *P* = 0.226). After VNS-*10*, total count (46 [± 5]; *P* < 0.001), macrophage count (29 [± 5]; *P* = 0.020), and neutrophil count (15 [± 6]; *P* = 0.002) were all reduced.

**Fig. 4:**
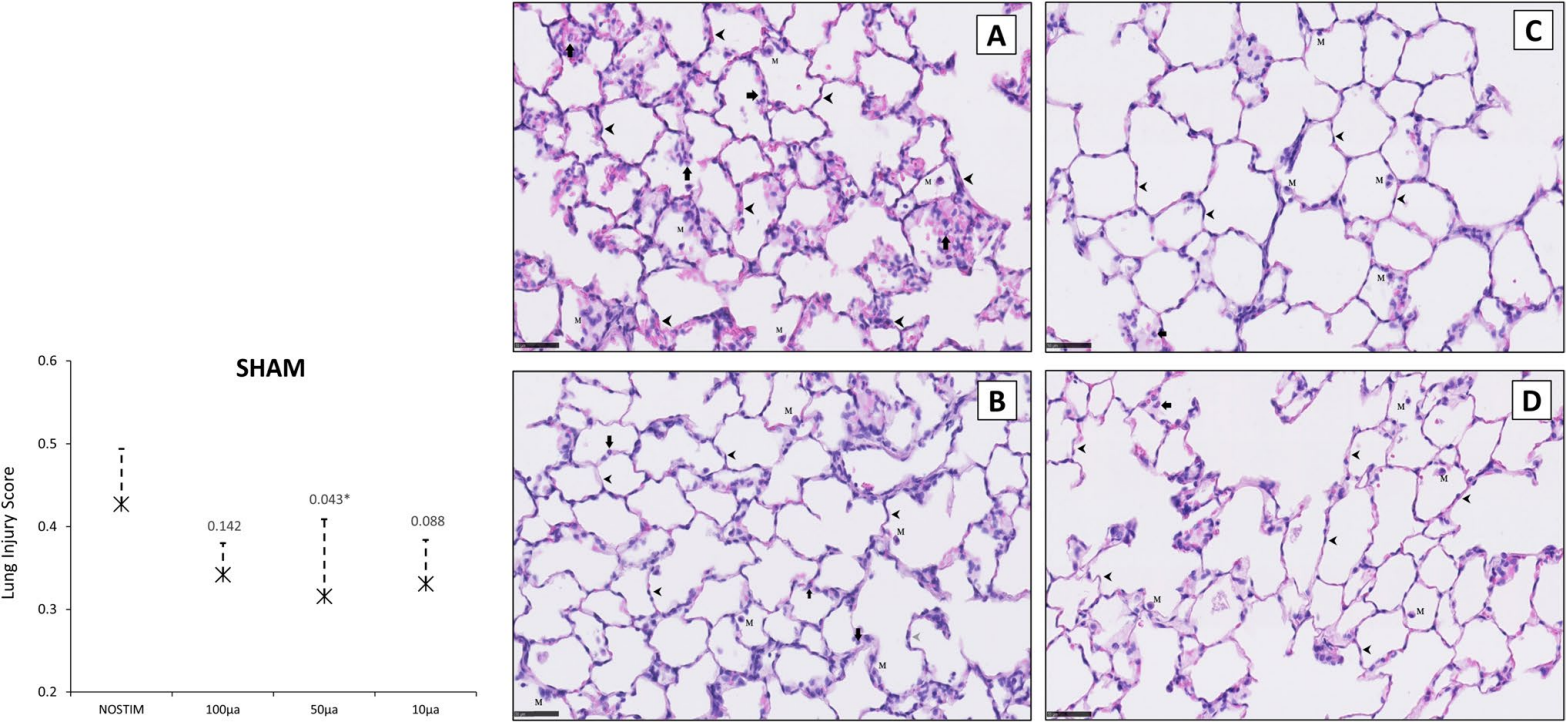
Histopathological lung injury score (LIS) in non-vagotomized rats. Histological sections of the lungs (H&E stained) at × 400 magnification (scale bar represents 50 µm). The NOSTIM group (**A**) showed some thickening of alveolar septa (*marked by arrowheads*) and neutrophil infiltration in the interstitial and alveolar spaces (*marked by arrows*). Alveolar mononuclear cell infiltration (e.g., plasma cells and macrophages) is observed (*marked by M*). LIS was not markedly affected by VNS with 100 µA (**B**). LIS was significantly reduced after VNS with 50 µA (**C**), whereas after 10 µA (**D**), this finding did not reach statistical significance. Note that the alveolar walls are thin and the alveoli contain occasional alveolar mononuclear cells (**C** and **D**). Values are means with standard deviation. A *P*-value of < 0.05 after post-hoc Dunn’s test was considered statistically significant. Abbreviations: NOSTIM, group without VNS; VNS, vagus nerve stimulation. *N* = 5 in all groups

Histopathological lung injury score (LIS) was 0.427 [SD ± 0.067] in the NOSTIM group (Fig. 4). LIS was similar after VNS-*100* (0.342 [± 0.038], *P* = 0.142) and after VNS-*10* (0.331 [± 0.053]; *P* = 0.088). In contrast, LIS was reduced after VNS-*50* (0.316 [± 0.093]; *P* = 0.043).

**Fig. 5:**
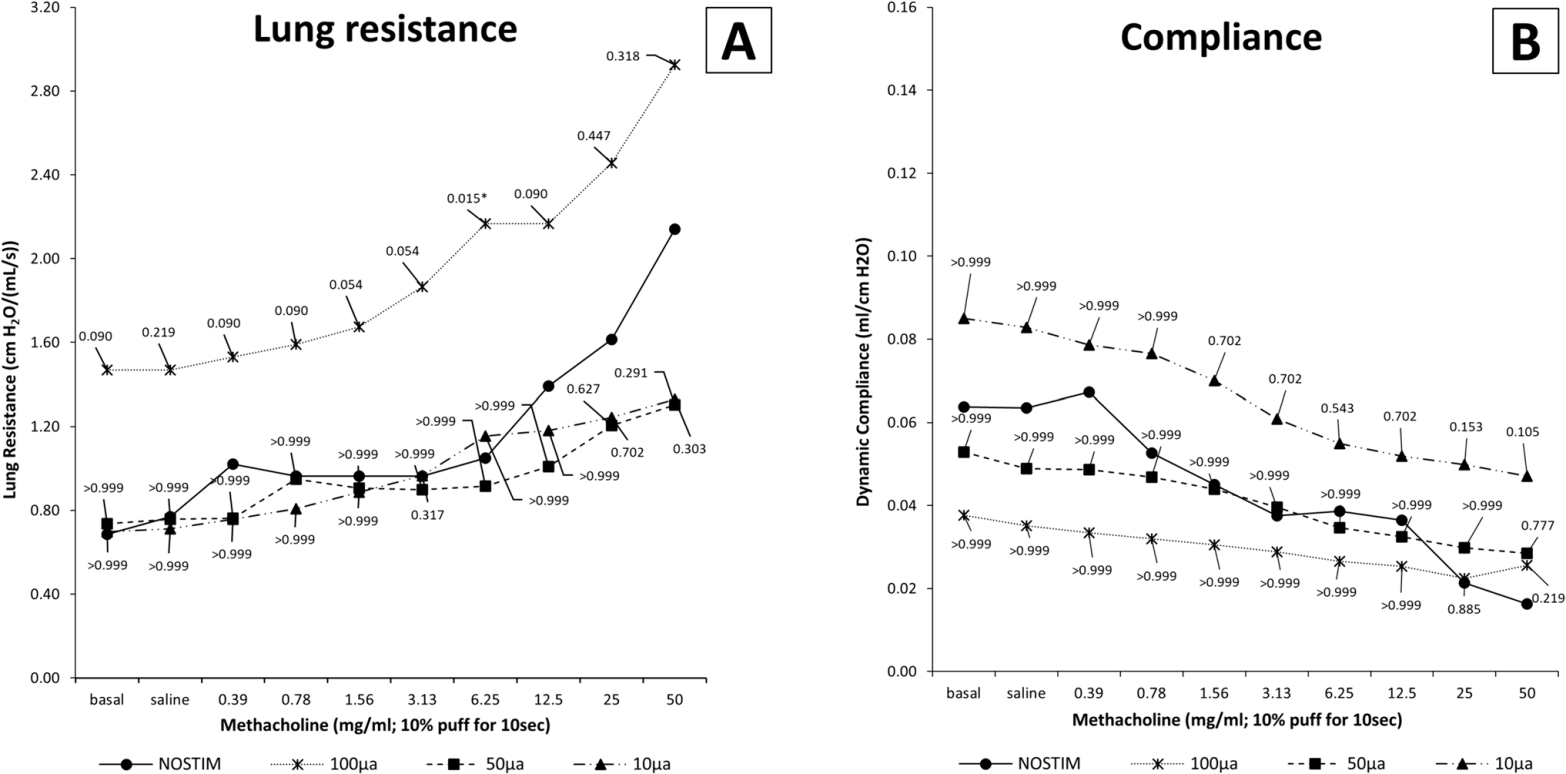
Pulmonary function in non-vagotomized rats. There were no statistically significant changes in **A** lung resistance or **B** dynamic compliance between the NOSTIM and VNS groups. Values are medians (interquartile range is not shown for clarity). A *P*-value of < 0.05 was considered statistically significant. *P*-values shown are Bonferroni-adjusted (multiplied by 3) as compared to NOSTIM. Abbreviations: NOSTIM, group without VNS; VNS, vagus nerve stimulation

Lung resistance in the NOSTIM group was median 0.687 (IQR 0.411–1.288) cm H2O/(mL/s) at baseline, 0.770 (IQR 0.341–1.492) after saline, and 2.140 (IQR 1.733–2.865) at the highest methacholine dose (50 mg/mL), as shown in Fig. 5A. Dynamic lung compliance was 0.064 (IQR 0.030–0.086) mL/cm H2O at baseline, 0.064 (IQR 0.030–0.082) after saline, and 0.016 (IQR 0.014–0.026) at the highest methacholine dose (Fig. 5B). There were no statistically significant differences in lung resistance or compliance between the NOSTIM and VNS groups.

In conclusion, in non-vagotomized rats, after VNS-*100*, the influx of neutrophils in BALF and LIS was not significantly affected. VNS-*50* reduced influx of neutrophils, and LIS was significantly reduced compared to NOSTIM. VNS-*10* reduced influx of neutrophils in BALF, whereas LIS was reduced but not statistically significantly. Pulmonary function was not affected by VNS. TNF-α and IL-6 were analyzed in plasma and serum but did not reach detectable systemic levels in any group.

### Vagotomy group

**Fig. 6:**
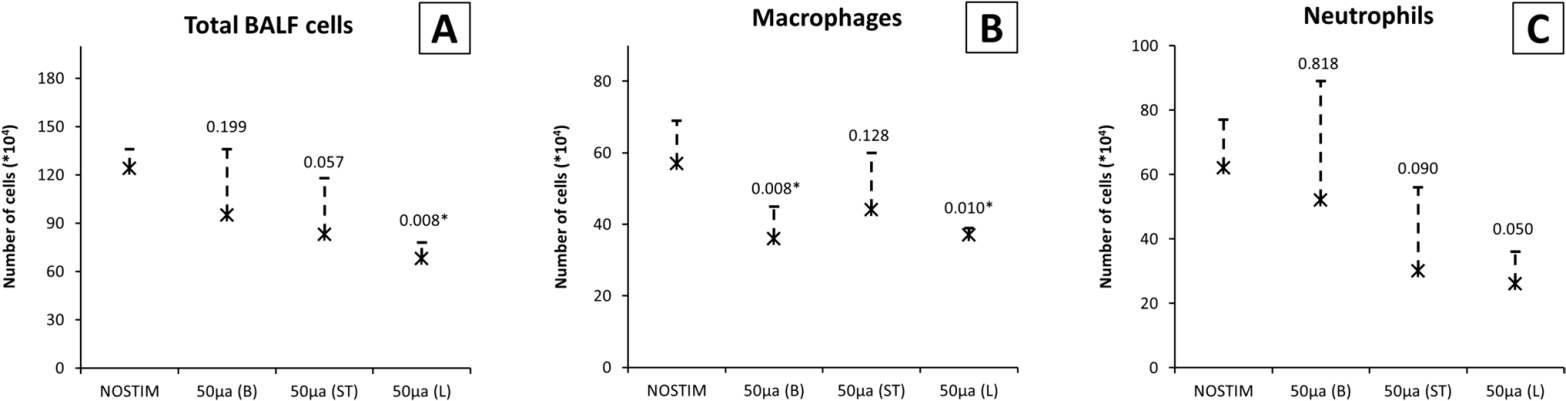
Number of inflammatory cells in BALF (× 10^4^) in the vagotomy group. **A** Total cell count, **B** macrophages, and **C** neutrophils. Bilateral VNS-*50-before* (50µA(B)) bilateral vagotomy and LPS (intratracheally) reduced the number of macrophages, but not neutrophils compared to NOSTIM. Bilateral VNS-*50-after* (50µA(ST); i.e., efferent vagal stumps) bilateral vagotomy reduced the number of neutrophils, but this did not reach statistical significance. VNS-*50-unilaterally* (50µA(L)) before ipsilateral (left) vagotomy reduced the influx of neutrophils and macrophages. Values are means with standard deviation. A *P*-value of < 0.05 after post-hoc Dunn’s test was considered statistically significant. Abbreviations: BALF, broncho-alveolar lavage fluid; LPS, lipopolysaccharide; NOSTIM, group without VNS; VNS, electrical vagus nerve stimulation

In the NOSTIM group, the total count of inflammatory cells (× 10^4^) in the BALF was mean 124 [SD ± 12], as shown in Fig. 6. Macrophage count (× 10^4^) was 57 [SD ± 12], and count of neutrophils (× 10^4^) was 62 [SD ± 15]. In the VNS-*50-before* group, total count of inflammatory cells (95 [± 41]; *P* = 0.199) and neutrophil count (52 [± 37]; *P* = 0.818) were similar compared with NOSTIM, whereas macrophage count was reduced (36 [± 9]; *P* = 0 0.008). In the VNS-*50-after* group, the total count of inflammatory cells (83 [± 35]; *P* = 0.057), macrophages (44 [± 16]; *P* = 0.128), and neutrophils (30 [± 26]; *P* = 0.090) was not significantly reduced. In the VNS-*50-unilaterally* group, the total count (68 [± 10]; *P* = 0.008) was reduced, as well as macrophage count 37 [± 2]; *P* = 0.010) and neutrophil count (26 [± 10]; *P* = 0.050).

**Fig. 7:**
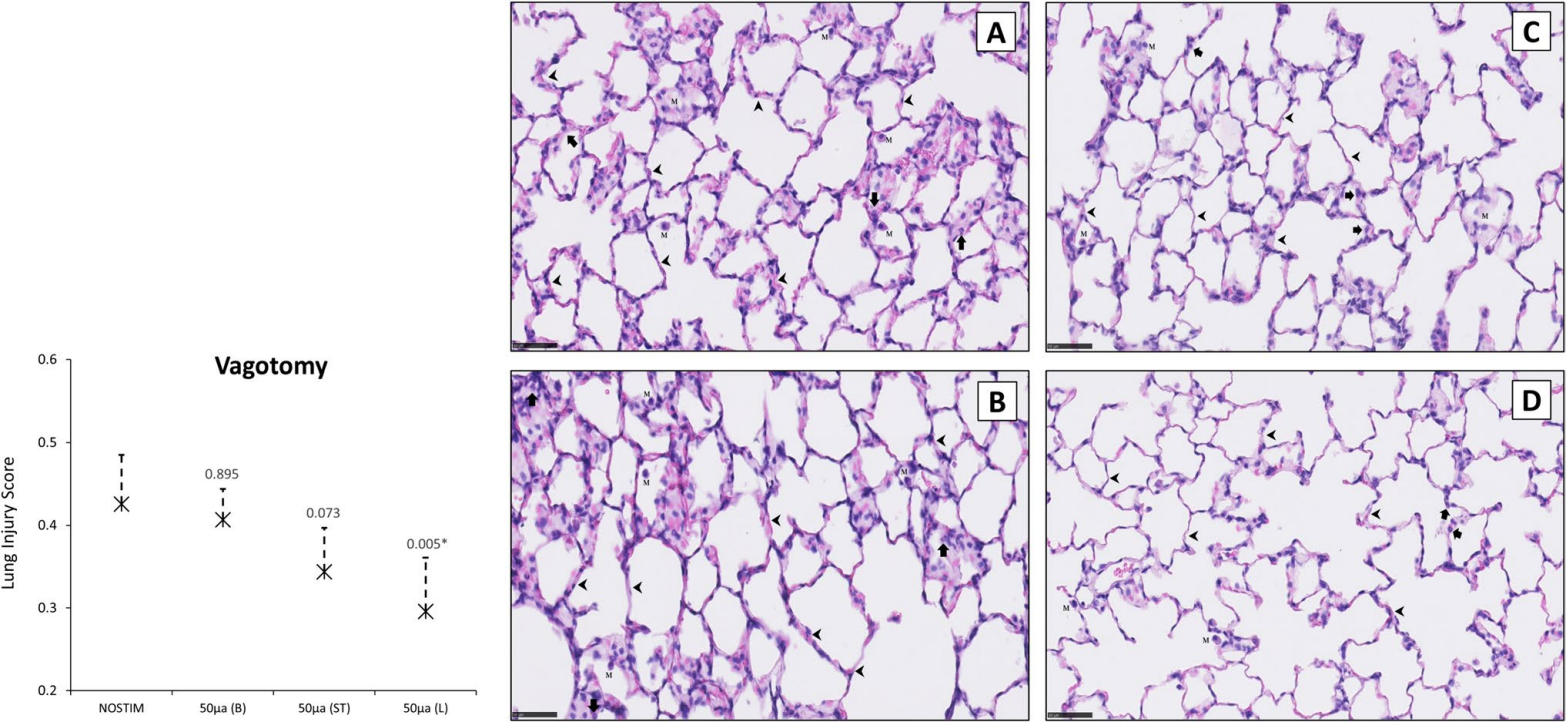
Histopathological lung injury score (LIS) in the vagotomy group. Histological sections of the lungs (H&E stained) at × 400 magnification (scale bar represents 50 µm). The NOSTIM group (**A**) showed some thickening of alveolar septa (*marked by arrowheads*) and neutrophil infiltration in the interstitial and alveolar spaces (*marked by arrows*). Alveolar mononuclear cell infiltration (e.g., plasma cells and macrophages) is observed (*marked by M*). LIS was not affected by VNS-*50-before* (50µA(B)) bilateral vagotomy (**B**). VNS-*50-after *(50µA(ST); i.e., efferent stumps) bilateral vagotomy reduced LIS, but not statistically significantly (**C**). VNS-*50-unilaterally *(50µA(L)) before ipsilateral (left) vagotomy significantly reduced LIS (**D**). Note that the alveolar walls are thin and the alveoli contain occasional alveolar mononuclear cells (**C** and **D**). Values are means with standard deviation. A *P*-value of < 0.05 after post-hoc Dunn’s test was considered statistically significant. Abbreviations: LPS, lipopolysaccharide; NOSTIM, group without VNS; VNS, electrical vagus nerve stimulation. *N* = 5 in all groups

Histopathological lung injury score (LIS) was 0.426 [SD ± 0.059] in the NOSTIM group (Fig. 7). In the VNS-*50-before* group, LIS was similar compared with NOSTIM (0.407 [± 0.037]; *P* = 0.895). VNS-*50-after* reduced LIS but not statistically significantly (0.344 [± 0.053]; *P* = 0.073). In contrast, LIS was significantly reduced (0.296 [± 0.065]; *P* = 0.005) compared with NOSTIM in the VNS-*50-unilaterally* group.

**Fig. 8:**
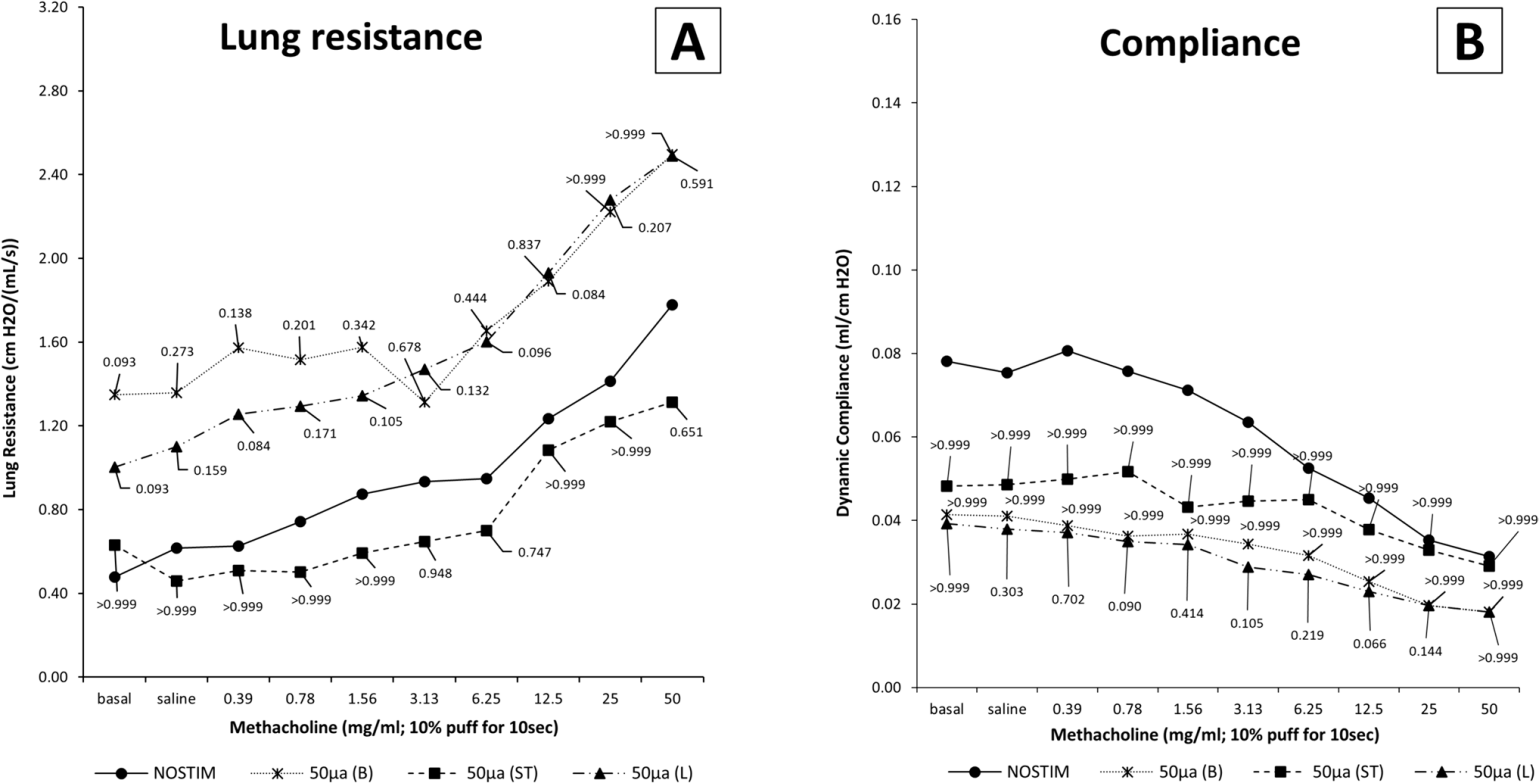
Pulmonary function in the vagotomy group. There were no statistically significant changes in (**A**) lung resistance or (**B**) dynamic compliance between the NOSTIM and VNS groups. Values are medians (interquartile range is not shown for clarity). A *P*-value of < 0.05 was considered statistically significant. *P*-values shown are Bonferroni-adjusted (multiplied by 3) as compared to NOSTIM. Abbreviations: NOSTIM, group without VNS; VNS, electrical vagus nerve stimulation; 50µA(B), VNS-*50-before* bilateral vagotomy; 50µA(ST), VNS-*50-after* bilateral vagotomy (i.e., efferent stumps); 50µA(L), VNS-*50-unilaterally* (left) before ipsilateral vagotomy

Lung resistance in the NOSTIM group was median 0.477 (IQR 0.286–0.929) cm H2O/(mL/s) at baseline, 0.618 (IQR 0.326–1.007) after saline, and 1.778 (IQR 1.403–2.582) at the highest methacholine dose, as shown in Fig. 8A. Dynamic lung compliance was 0.0647 (IQR 0.035–0.141) mL/cm H2O at baseline, 0.066 (IQR 0.038–0.132) after saline, and 0.031 (IQR 0.017–0.042) at the highest methacholine dose (Fig. 8B). There were no statistically significant differences in lung resistance or compliance between the NOSTIM and VNS groups.

In conclusion, in vagotomized rats, VNS-*50-before* bilateral vagotomy did not affect the influx of neutrophils or LIS. VNS-*50-after* bilateral vagotomy (i.e., efferent vagal stumps) reduced the influx of neutrophils and LIS, but this finding did not reach statistical significance. VNS-*50-unilaterally (*left) before ipsilateral vagotomy reduced both the influx of neutrophils and LIS. There were no differences in pulmonary function between groups. Plasma and serum TNF-α and IL-6 did not reach detectable systemic levels in any group.

## Discussion

In this experimental study, it was shown that the influx of inflammatory cells in rat lungs, induced by intra-tracheal administration of LPS, was diminished after (electrical) VNS. This finding was similar to previous animal models showing that (electrical) stimulation of the vagus nerve protects rodents against endotoxemia by downregulating inflammatory responses [15–18, 26, 31, 34–36]. Although, in the current study, the effect on neutrophil influx was unequivocal after VNS was delivered with lower electrical current (i.e., 50 and 10 µA), its effect was diminished when applying 100 µA. This indicates that there may also be harmful effects due to excessive (electrical) stimulation, which we hypothesized could be the result of an exaggerated release of acetylcholine leading to bronchoconstriction [12, 37–40].

The observed effect of VNS on the influx of inflammatory cells was ambiguous in vagotomized rats. In rats that received bilateral VNS-*50-before* performing a bilateral vagotomy, the influx of neutrophils was not affected. Bilateral VNS-*50-after* bilateral vagotomy (i.e., stimulating the efferent vagal stumps) lowered the influx of neutrophils, but this finding did not reach statistical significance. It is not unexpected that VNS failed to inhibit the influx of neutrophils in bilaterally vagotomized rats, since both afferent and efferent vagal activity is blocked, thereby interrupting the anti-inflammatory pathways of the vagus [29, 30, 41–44]. Although this is the first study to investigate the effect of electrical VNS on local inflammation (i.e., lung) in vagotomized rats, a study by Komegae and co-workers investigated its effect on systemic inflammation. It was reported that selective electrical stimulation of vagal afferents (by crushing the vagus efferents) reflexively modulated systemic inflammation, whereas VNS failed to inhibit systemic inflammation after vagal afferent feedback was blocked due to bilateral cervical vagotomy [29]. Hence, based on the results in the current study, it is likely that the afferent pathway is also imperative in regulating inflammation in the lung. This was further supported by the findings in unilaterally vagotomized rats. Specifically, VNS-*50-unilaterally* left before ipsilateral vagotomy reduced the influx of neutrophils. Taking into account that the BALF in which neutrophils were counted was taken from the contralateral side (i.e., right lung), the simplest interpretation of this finding is that stimulating vagal afferents on one side activates both ipsilateral and contralateral vagal anti-inflammatory pathways [29]. An alternative possibility could be that a proportion of the pulmonary vagal branches on one side also course towards the contralateral side. This has been previously shown in a cadaveric study; however, whether this is true for rats is currently unknown [12, 13].

Histopathological lung injury (LIS) is also a key pathophysiologic feature in acute lung injury [33]. In non-vagotomized rats, it was shown that VNS with 50 and 10 µA attenuated LIS, although this finding only reached statistical significance in the 50 µA group. The short timeline of the experiments likely affected the observed histological changes as neutrophil infiltration did not yet peak in response to the LPS challenge [45, 46]. In vagotomized rats, VNS-*50-before* bilateral vagotomy did not influence LIS. VNS-*50-after* bilateral vagotomy attenuated LIS, but not statistically significantly, whereas VNS-*50-unilaterally* left before ipsilateral vagotomy significantly reduced LIS (i.e., left lung). These results show that, similar to previous studies of systemic endotoxemia [15–18, 26, 29, 31, 34, 35, 47], local inflammation in rat lungs is also attenuated by anti-inflammatory pathways of the vagus nerve.

The current data might have potential clinical implications. Performing the vagotomy distal to the caudal most large pulmonary branch after the lymphadenectomy, or electrically stimulating these branches during an esophagectomy, could potentially attenuate the postoperative inflammatory response similar to disorders involving the innate immune system, such as rheumatoid arthritis and Crohn’s disease [21–24]. However, it remains unclear what the clinical applicability of VNS during esophagectomy is due to potential adverse cardiac events, but also other unwanted side effects such as hoarseness, dyspnea, and dysphagia as a result of vocal cord paralysis [39, 48–50]. During esophagectomy, pulmonary branches of the right vagus nerve are identified. Stimulation of these branches may especially increase the risk of (cardiac) side effects (i.e., bradycardia or asystole), since the heart is primarily innervated by the right vagus nerve. However, a study by Wong et al. reported that VNS may be used for continuous intraoperative nerve monitoring to aid in lymph node dissection along the recurrent laryngeal nerves [51]. It remains to be elucidated whether the electrical signal for nerve monitoring is sufficient to activate the CAIP.

On the other hand, the anti-inflammatory input is only partly attributable to (local) stimulation and it has been shown that—despite the lack of a direct connection with the vagus nerve—the spleen also plays a pivotal role in the neural anti-inflammatory pathway [21, 26, 30, 41–44, 52]. Studies in animal models suggest that the vagus nerve interacts with the spleen through adrenergic neurons located in the celiac ganglion, which release norepinephrine via the splenic nerve [30, 44, 52]. In a catecholamine-dependent fashion, the production of pro-inflammatory mediators by splenic macrophages is inhibited, thereby attenuating inflammatory responses. Splenic nerve stimulation during an esophagectomy could be more readily performed and less challenging, but with similar effects on the postoperative inflammatory response. A recent feasibility study conducted for the first time in patients undergoing MIE demonstrated that applying electrical current via a cuff around the splenic arterial neurovascular bundle—which is exposed as part of the procedure—is safe, feasible, and may have an effect on the postoperative inflammatory response [53]. These findings suggest that neuromodulation of the splenic arterial neurovascular bundle may offer a new approach in immunomodulatory therapy, but large randomized trials are needed to support this hypothesis [53].

We acknowledge that the findings of this randomized animal study should be interpreted within the context of several limitations. Although a relatively large total population of rats was used, groups were small since there were several research models to test our hypotheses. This may be subject to imprecision and inaccuracies (outliers). Therefore, to adjust for multiple comparisons, the post-hoc Dunn’s and Bonferroni tests were used for normal and non-normal distributions, respectively, but these may be overly conservative [54]. A strength of the current study is that three of the four key domains of acute lung injury in experimental models (i.e., inflammatory cells, histopathology, and physiological function) were met, while we did not have the tools to assess alterations of the Alveolar–Capillary barrier (e.g., lung wet-to-dry weight ratio) [33].

In conclusion, the results of this study indicate that electrical VNS inhibited LPS-induced influx of neutrophils in the lung and may limit histopathological lung injury, but its effect is dependent on (partially) intact vagus nerves. It is suggested that the level of the vagotomy during esophagectomy may influence postoperative pulmonary outcomes. The vagus nerve could potentially be artificially stimulated to reduce postoperative pulmonary complications after esophagectomy.

## Supplementary Information

Below is the link to the electronic supplementary material.

Supplementary file1 (DOCX 303 KB)

## Data Availability

All data will be made available by the corresponding author upon request.
