# Supplementary material for: Electrical vagus nerve stimulation is a promising approach to reducing pulmonary complications after an esophagectomy: an experimental rodent model
Source: Immunol Res. 2024 Jul 31;72(6):1247–58. doi: 10.1007/s12026-024-09523-3 (PMC11618150; doi:10.1007/s12026-024-09523-3)
Supplement: Supplementary file 1 — Supplementary file1 (DOCX 303 KB) [file 12026_2024_9523_MOESM1_ESM.docx]

**SUPPLEMENTARY FILE**


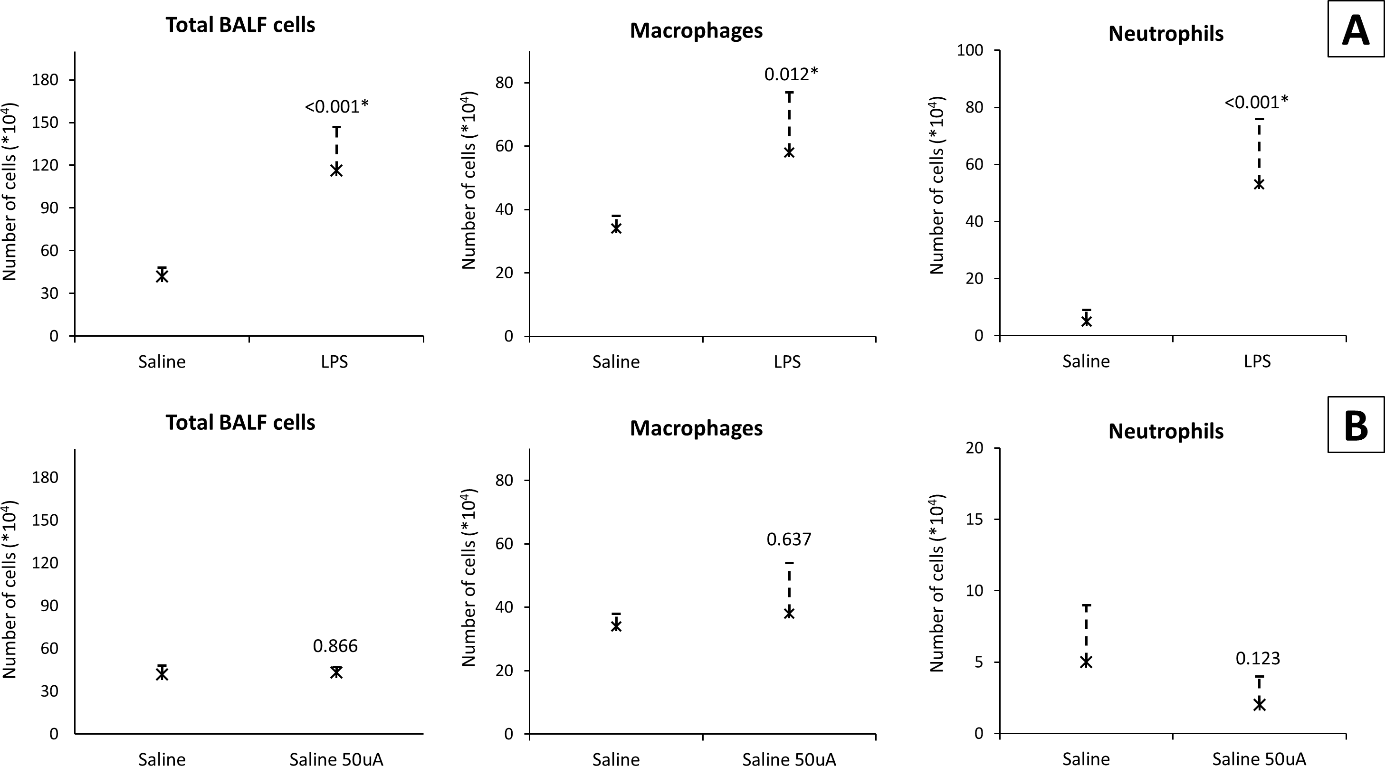


**Supplementary Figure 1. Number of inflammatory cells in BALF (*10^4^) in non-vagotomized rats.** In **(a)** total cell count, macrophages and neutrophils between saline (0.9% NaCl) and intratracheal LPS (0.3 µg/kg) are presented. In **(b)** total cell count, macrophages and neutrophils between saline without stimulation and saline with bilateral electrical VNS at 50μA. Values are means with standard deviation. A p-value of < 0.05 was considered statistically significant. Abbreviations: BALF *broncho-alveolar lavage fluid;* LPS *lipopolysaccharide*; VNS *electrical vagus nerve stimulation*.


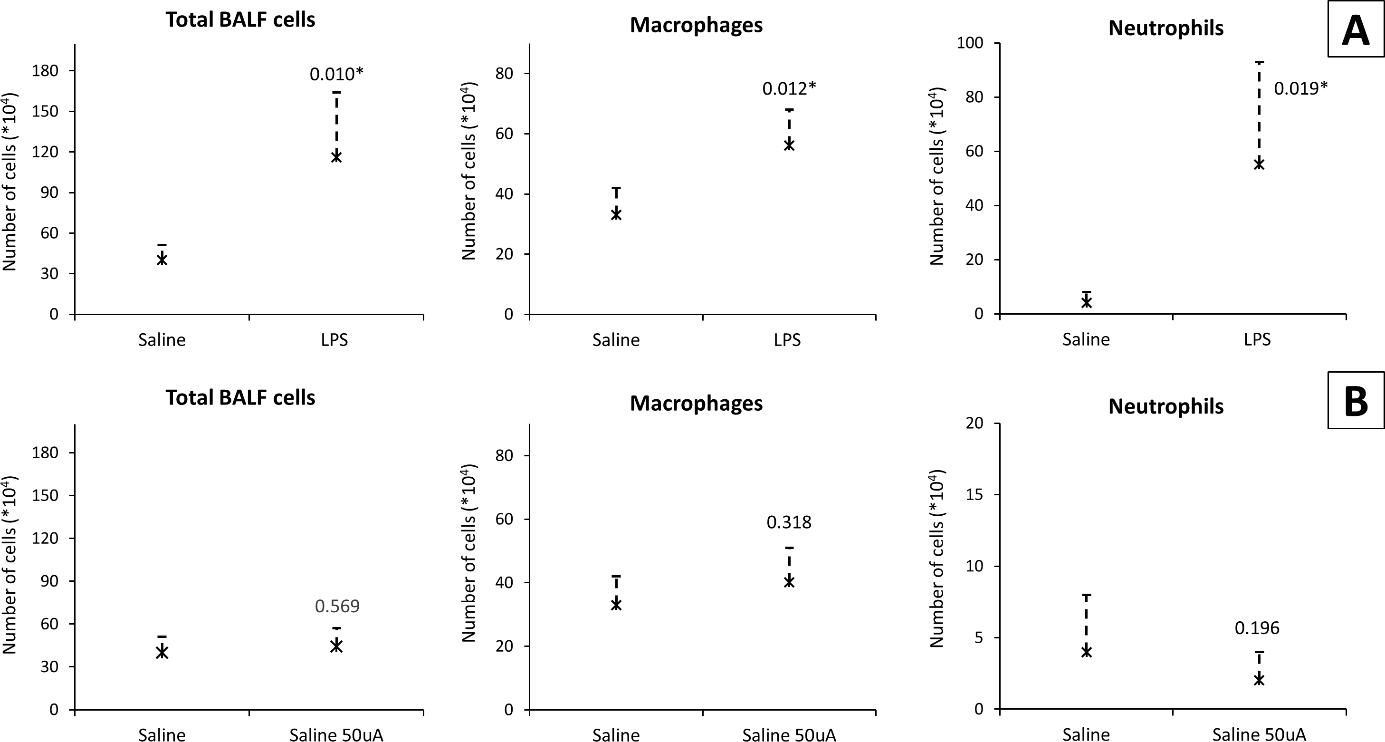


**Supplementary Figure 2. Number of inflammatory cells in BALF (*10^4^) in rats after bilateral vagotomy.** In **(a)** total cell count, macrophages and neutrophils between saline (0.9% NaCl) and intratracheal LPS (0.3 µg/kg) are presented. In **(b)** total cell count, macrophages and neutrophils between saline without stimulation and saline with bilateral electrical VNS at 50μA prior to vagotomy. Values are means with standard deviation. A p-value of < 0.05 was considered statistically significant. Abbreviations: BALF *broncho-alveolar lavage fluid;* LPS *lipopolysaccharide*; VNS *electrical vagus nerve stimulation*.
